# Loss of muscleblind-like 1 promotes invasive mesenchyme formation in endocardial cushions by stimulating autocrine TGFβ3

**DOI:** 10.1186/1471-213X-12-22

**Published:** 2012-08-06

**Authors:** Kathryn E LeMasters, Yotam Blech-Hermoni, Samantha J Stillwagon, Natalie A Vajda, Andrea N Ladd

**Affiliations:** 1 Department of Cell Biology, Lerner Research Institute, Cleveland Clinic, 9500 Euclid Ave., Mail code NC10, Cleveland, OH, 44195, USA

**Keywords:** Muscleblind-like 1, Alternative splicing, TGFβ, Endocardial cushions, Epithelial-mesenchymal transition, Cell invasion

## Abstract

**Background:**

Valvulogenesis and septation in the developing heart depend on the formation and remodeling of endocardial cushions in the atrioventricular canal (AVC) and outflow tract (OFT). These cushions are invaded by a subpopulation of endocardial cells that undergo an epithelial-mesenchymal transition in response to paracrine and autocrine transforming growth factor β (TGFβ) signals. We previously demonstrated that the RNA binding protein muscleblind-like 1 (MBNL1) is expressed specifically in the cushion endocardium, and knockdown of MBNL1 in stage 14 embryonic chicken AVC explants enhances TGFβ-dependent endocardial cell invasion.

**Results:**

In this study, we demonstrate that the effect of MBNL1 knockdown on invasion remains dependent on TGFβ3 after it is no longer required to induce basal levels of invasion. TGFβ3, but not TGFβ2, levels are elevated in medium conditioned by MBNL1-depleted AVC explants. TGFβ3 is elevated even when the myocardium is removed, indicating that MBNL1 modulates autocrine TGFβ3 production in the endocardium. More TGFβ3-positive cells are observed in the endocardial monolayer following MBNL1 knockdown. Addition of exogenous TGFβ3 to AVC explants recapitulates the effects of MBNL1 knockdown. Time course experiments demonstrate that knockdown of MBNL1 induces precocious TGFβ3 secretion, and early exposure to excess TGFβ3 induces precocious invasion. MBNL1 expression precedes TGFβ3 in the AVC endocardium, consistent with a role in preventing precocious autocrine TGFβ3 signaling. The stimulatory effects of MBNL1 knockdown on invasion are lost in stage 16 AVC explants. Knockdown of MBNL1 in OFT explants similarly enhances cell invasion, but not activation. TGFβ is necessary and sufficient to mediate this effect.

**Conclusions:**

Taken together, these data support a model in which MBNL1 negatively regulates cell invasion in the endocardial cushions by restricting the magnitude and timing of endocardial-derived TGFβ3 production.

## Background

Epithelial-mesenchymal transition (EMT) is a critical process in the formation of the valves and septa of the heart. Prior to the onset of EMT, endocardial cushions form in the atrioventricular canal (AVC) and outflow tract (OFT) regions when localized deposition of extracellular matrix separates the endocardial lining from the myocardial wall of the heart tube. In response to inductive signals from the adjacent myocardium, a subset of endocardial cells within the AVC and OFT cushions undergoes EMT and invades the cushion matrix [1-4]. Fusion and remodeling of the cellularized cushions in the AVC gives rise to the mitral and tricuspid valves, and in the OFT to the aortic and pulmonary valves. Dysregulation of EMT or subsequent remodeling of the cushion mesenchyme can cause valve and septal malformations [5], which can range from mild to life threatening.

Endocardial cell EMT can be separated into two distinct events: activation, in which cell polarity and cell-cell contacts are lost, and invasion, in which the transformed mesenchymal cells migrate into the matrix. In the chick, both steps are induced by members of the transforming growth factor β (TGFβ) family. TGFβ2 is required for activation, while TGFβ3 is required for invasion [6,7]. TGFβ2 and TGFβ3 are both expressed in the underlying myocardium prior to mesenchyme formation [6,8,9]. In contrast, TGFβ3 is expressed in the cushion endocardium only during stages of active mesenchyme formation [6,8,9]. Although secreted signals from the AVC and OFT myocardium have been shown to induce endocardial cell EMT [1-4], it has also been demonstrated that an autocrine TGFβ3 signal is required for endocardial cell transformation into invasive mesenchyme [9,10]. This autocrine signal likely provides amplification or propagation of TGFβ3 signaling within the endocardium, perhaps activated by the myocardium.

Muscleblind-like 1 (MBNL1) is a known regulator of pre-mRNA alternative splicing in the developing heart, driving fetal-to-adult transitions in alternative splicing [11,12]. MBNL1 has also recently been shown to regulate the stability of some mRNA targets [13]. We previously reported that MBNL1 is differentially expressed in the embryonic heart during endocardial cushion development [14]. Within the endocardial cushions, MBNL1 is highly expressed in the endocardium, but is low or absent from the underlying myocardium and invaded mesenchymal cells. In contrast, outside of the cushion regions MBNL1 is found in the myocardium but not the endocardium. Knockdown of MBNL1 in Hamburger and Hamilton (H&H) stage 14 embryonic chicken AVC explants with either of two independent siRNAs enhanced invasion, whereas a control non-targeting siRNA and an siRNA directed against an unrelated splicing factor had no effect [14]. We demonstrated that this enhancement of invasion in response to MBNL1 knockdown is TGFβ3-dependent, but the basis for this dependence had not yet been determined.

In this study, we investigate the relationship between MBNL1 and TGFβ3 in the AVC and OFT cushions. We provide evidence that knockdown of MBNL1 leads to precocious and elevated endocardial-derived TGFβ3 levels in AVC explants, and that early exposure to excess TGFβ3 leads to precocious and elevated levels of mesenchymal cell invasion. Likewise, TGFβ is necessary and sufficient to stimulate excess invasion in response to MBNL1 depletion in OFT explants. Taken together, these data support a model in which MBNL1 acts as a negative regulator of endocardial cushion EMT by restricting the timing and amount of autocrine TGFβ3 production in the AVC and OFT endocardium.

## Results

### Enhancement of cell invasion in the AVC by MBNL1 knockdown is dependent on TGFβ3

**Figure 1:**
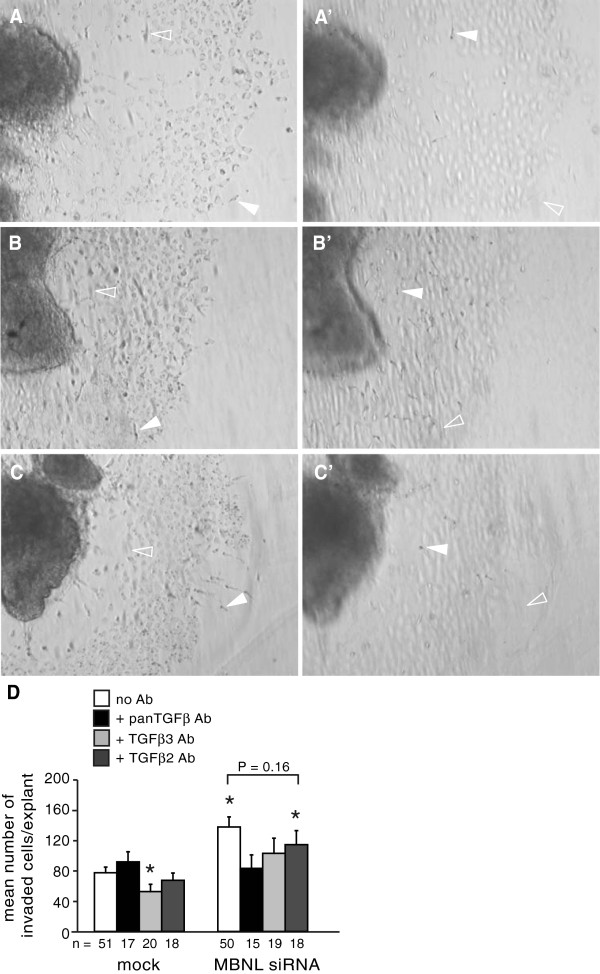
**Effects of MBNL1 knockdown on cell invasion remain dependent on TGFβ3 after induction of EMT.** Stage 14 AVC explants were transfected with or without MBNL1 siRNA. Anti-TGFβ antibodies or medium alone were added at 18 hours. Representative images of explants that were mock-transfected plus medium alone (**A**, **A’**), MBNL1 siRNA-transfected plus medium alone (**B**, **B’**), and MBNL1 siRNA-transfected plus anti-TGFβ3 antibody (**C**, **C’**) were taken in the focal plane of the surface of the gel (**A**, **B**, **C**) and within the gel (**A’**, **B’**, **C’**) to show invaded mesenchymal cells. Filled arrowheads indicate cells in the focal plane, whereas open arrowheads indicate the same cells out of focus in a different plane. (**D**) The number of invaded cells was counted at 38 hrs. The mean number of invaded cells per explant + the standard error of the mean is shown. Inhibition of TGFβ signaling has little effect on the basal level of cell invasion in mock-transfected explants. Inhibition with a pan-TGFβ or TGFβ3-specific antibody blocked the enhancement of cell invasion by MBNL1 knockdown, whereas inhibition with a TGFβ2-specific antibody did not. An asterisk indicates a significant difference from mock-transfected explants without antibody treatment (P ≤ 0.05).

TGFβ3 is required for AVC mesenchyme invasion [6,7,9,10]. This requirement is not abrogated by loss of MBNL1. We previously demonstrated that the enhancement of cell invasion induced by knockdown of MBNL1 in stage 14 AVC explants can be blocked by inhibitory antibodies against TGFβs, in particular TGFβ3 [14]. In those experiments the TGFβ-neutralizing antibodies were added at six hours. At this time point, blocking TGFβ effectively abolishes EMT in control as well as siRNA-treated explants. It is therefore unclear whether MBNL1 acts through TGFβ3, or merely that TGFβ3 signaling is required to initiate the process of invasion, which is then acted on by MBNL1 at a later step. To address this, we added anti-TGFβ neutralizing antibodies to mock- or MBNL1 siRNA-treated stage 14 AVC explants eighteen hours after explantation (Figure 1). At this time, addition of a pan anti-TGFβ antibody that inhibits TGFβ1, -2, and −3 had no effect on invasion in control explants, indicating that the requirement for TGFβ signaling to induce basal levels of invasion has passed, presumably because sufficient inductive signal has already been received. This is consistent with a previous study that showed anti-TGFβ3 antibodies have progressively less inhibitory effect on invasion in AVC explants taken from progressively later stages, suggesting that over time an increasing number of cushion endocardial cells pass the critical requirement for TGFβ3 [6]. Inhibition of TGFβ did abrogate the increase in invasion induced by anti-MBNL1 siRNA treatment, however, suggesting that the effects of MBNL1 knockdown remain dependent on TGFβ signaling. An anti-TGFβ3 inhibitory antibody likewise reduced invasion to control levels in MBNL1 siRNA-treated explants. The requirement for TGFβ3 is specific, as levels of invasion remained elevated in MBNL1-depleted explants in the presence of an anti-TGFβ2 inhibitory antibody.

### Knockdown of MBNL1 enhances autocrine TGFβ3 in the AVC endocardium

**Figure 2:**
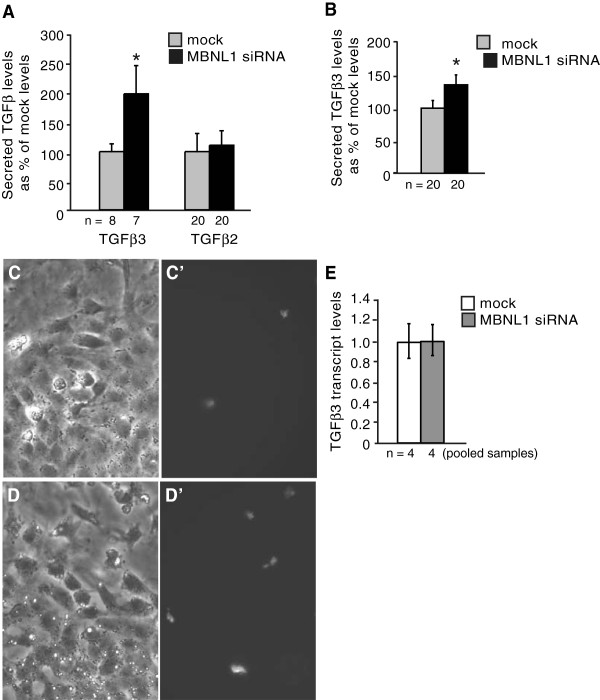
**Loss of MBNL1 leads to elevated endocardial-produced TGFβ3 levels.** Stage 14 AVC explants were transfected with or without MBNL1 siRNA. At 18 hrs medium was added to intact explants (**A**) or explants where the myocardium has been removed (**B**). Supernatants were collected after 20 hrs of conditioning and assayed by ELISA. The mean levels of secreted TGFβ proteins as percentages of the mock level + the standard errors of the means are shown. (**A**) TGFβ3, but not TGFβ2, levels were elevated in the supernatants from MBNL1 siRNA-treated explants. (**B**) TGFβ3 levels were elevated in supernatants from MBNL1 siRNA-treated explants in which the myocardium has been removed prior to conditioning. Immunofluorescence with an anti- TGFβ3 antibody was also performed on mock- (**C**, **C’**) or MBNL1 siRNA-transfected (**D**, **D’**) stage 14 AVC explants fixed after 38 hrs in culture. Bright field images of the endocardial monolayer in mock-transfected (**C**) and MBNL1 siRNA-transfected (**D**) explants correspond to immunofluorescence images for the same fields (**C’**, **D’**). (**E**) Stage 14 AVC explants were transfected with or without MBNL1 siRNA. After 38 hrs in culture, the myocardium was removed, RNA was harvested, and *TGFβ3* levels were evaluated by real-time RT-PCR. *TGFβ3* transcript levels did not change in response to MBNL1 knockdown in AVC endocardial cells. Error bars denote 95% confidence intervals. An asterisk indicates a significant difference from mock-transfected explants (P ≤ 0.05).

One possible explanation for the continued dependence on TGFβ3 for the enhancement of invasion in MBNL1 siRNA-treated explants is that loss of MBNL1 leads to an increase in TGFβ3. To determine whether TGFβ3 levels are affected by MBNL1 knockdown, secreted TGFβ protein levels were compared in conditioned media collected from mock- and MBNL1 siRNA-treated stage 14 AVC explants by ELISA (Figure 2A). Medium from siRNA-treated explants contained approximately twice as much TGFβ3 as medium from control explants. In contrast, TGFβ2 levels were similar in media from MBNL1-depleted and control explants.

Within the AVC, TGFβ3 is expressed in the myocardium before, during, and after EMT takes place, but is not expressed in active form in the endocardium until the onset of invasion [6,8,9]. MBNL1 is highly expressed in the AVC endocardium, but is not detected in the underlying myocardium [14]. To ascertain whether the increase in TGFβ3 secretion in MBNL1-depleted AVC explants is endocardially-derived, TGFβ3 levels were compared in conditioned media collected from mock- and MBNL1 siRNA-treated stage 14 AVC explants in which the myocardium was removed (Figure 2B). As in intact explants, secreted TGFβ3 levels were elevated in MBNL1-depleted AVC explants lacking the myocardium, indicating loss of MBNL1 has a cell-autonomous effect on autocrine TGFβ3 production in the endocardium. It may be noted that TGFβ3 levels are elevated in MBNL1-depleted AVC endocardia to a lesser extent than in intact MBNL1-depleted AVC explants. This could indicate a contribution of increased paracrine TGFβ3 production resulting from a non-cell-autonomous effect of MBNL1-depleted endocardium on the myocardium. This discrepancy may also result from a reduction in the endocardial cell population in the absence of the myocardium. Cardiomyocyte-conditioned medium has been shown to induce proliferation in AVC endocardial monolayers [9]. Indeed, the size of the endocardial monolayer, number of activated endocardial cells, and amount of invaded mesenchyme were significantly reduced in both control and siRNA-treated AVC endocardia relative to similarly treated intact AVC explants (data not shown).

To further confirm that TGFβ3 levels are elevated in endocardial cells following MBNL1 knockdown, immunofluorescence was performed on mock- and siRNA-transfected stage 14 AVC explants using an anti-TGFβ3 antibody. In control explants, TGFβ3-positive cells were detected within the endocardial monolayer (Figure 2C). This is consistent with the detection of active TGFβ3 in scattered cells within the AVC endocardium during stages of active EMT [8]. In agreement with our ELISA data, more TGFβ3-positive cells were detected within the endocardial monolayers of MBNL1-depeleted explants than controls (Figure 2D).

In some cases, MBNL1 binding to the 3' untranslated region of a target mRNA can promote mRNA decay, and loss of MBNL1 can increase transcript half-life [13]. To determine whether the increase in TGFβ3 levels following MBNL1 knockdown is due to an increase in *TGFβ3* transcripts, RNA was collected from the non-myocardial cells in mock and MBNL1 siRNA-treated stage 14 AVC explants and *TGFβ3* levels were measured by real time RT-PCR (Figure 2E). Despite a doubling in secreted TGFβ3 protein levels, steady state *TGFβ3* transcript levels exhibited no difference in MBNL1-depleted explants relative to controls. This indicates that the effects of MBNL1 on TGFβ3 levels are post-transcriptional, acting at the level of protein production, stability, or processing.

### Addition of exogenous TGFβ3 is sufficient to recapitulate the effects of MBNL1 knockdown in AVC explants

**Figure 3:**
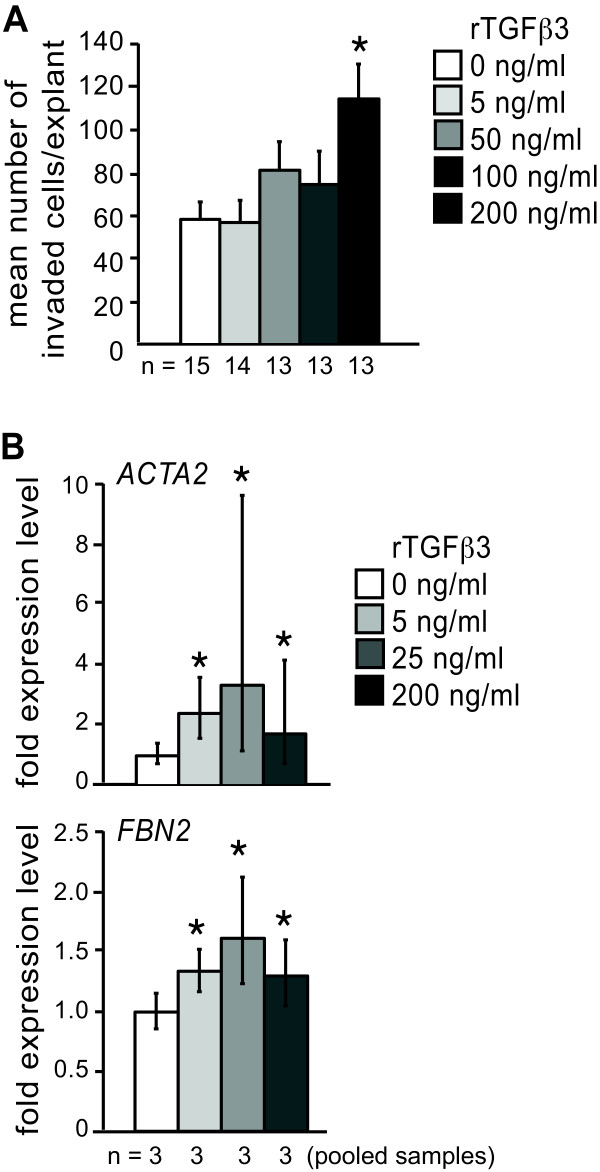
**Addition of exogenous TGFβ3 recapitulates the effects of MBNL1 knockdown.** Stage 14 AVC explants were treated with M199 supplemented with 0 to 200 ng/ml recombinant TGFβ3 at 6 hours. (**A**) The number of invaded cells was counted at 38 hrs. The mean number of invaded cells per explant + the standard error of the mean is shown. Addition of rTGFβ3 induced an increase in cell invasion, comparable at the highest dose tested to that seen following MBNL1 siRNA treatment. (**B**) The myocardium was removed and total RNA was harvested at 38 hrs. Expression of the mesenchymal markers *ACTA2* and *FBN2* were measured by real-time RT-PCR. Error bars denote 95% confidence intervals. Although the extent of *ACTA2* and *FBN2* up-regulation was somewhat variable, both markers were elevated in each of the pooled samples for all three doses (individual data not shown). An asterisk indicates a significant difference from 0 ng/ml rTGFβ3 (P ≤ 0.05).

TGFβ3 is necessary and sufficient to induce invasion in responsive AVC endocardial monolayers in the absence of the myocardium [9,10]. Supplementation with additional exogenous TGFβ3 protein has been shown to induce increased expression of mesenchymal markers in intact chick AVC explants [6], but whether or not exposure to extra TGFβ3 would also lead to increased levels of invasion has not been reported. To determine whether the presence of extra TGFβ3 protein is sufficient to enhance cell invasion, stage 14 AVC explants were treated with different doses of recombinant TGFβ3 protein. A trend towards increased cell invasion was observed, nearly doubling at the highest dose tested (Figure 3A).

Although the maximum level of invasion observed is comparable to that seen in MBNL1-depleted explants, the dose required to produce this effect (200 ng/ml) is one to two orders of magnitude higher than what has been shown to induce mesenchymal marker expression in AVC explants and endocardial monolayers [6,15]. To evaluate the induction of mesenchymal marker expression, total RNA was harvested from non-myocardial cells of stage 14 AVC explants following rTGFβ3 treatment over a similar dose range. Transcript levels for smooth muscle β-actin (*ACTA2*) and fibrillin 2 (*FBN2*), two EMT markers known to be induced by TGFβ3 [6,15], were assessed by real-time RT-PCR (Figure 3B). Both *ACTA2* and *FBN2* transcripts were induced at all doses tested. This indicates that higher doses of TGFβ3 are necessary to induce invasion than to activate mesenchymal gene expression, and that the expression of mesenchymal genes is not sufficient to drive invasion. Consistent with this, Nakajima and colleagues previously reported a dose-dependent induction of smooth muscle β-actin protein-positive cells by 2 to 20 ng/ml rTGFβ3 in AVC endocardial monolayers without acquisition of an invasive phenotype [15].

### Loss of MBNL1 induces precocious TGFβ3, and early exposure to excess TGFβ3 induces precocious invasion

**Figure 4:**
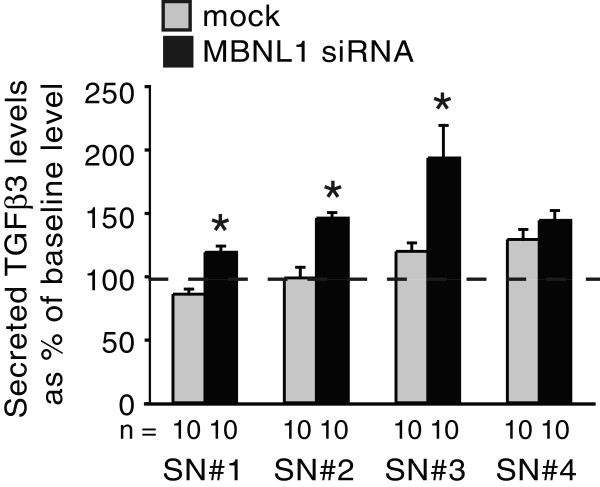
**Knockdown of MBNL1 induces precocious TGFβ3 secretion.** Stage 14 AVC explants were transfected with or without MBNL1 siRNA. After a boost at 6 hrs, medium was added to the explants. After 12 hrs, the conditioned medium was removed and fresh medium was added. This was repeated twice more, and all supernatants were assayed by ELISA. The mean levels of secreted TGFβ3 are shown as a percentage of the baseline level in the medium + the standard errors of the means. TGFβ3 levels in supernatants conditioned by mock-treated explants did not rise above the baseline level found in M199+ alone until 24–36 hrs after the boost. Supernatants from MBNL1 siRNA-treated explants, however, had increased levels of TGFβ3 within the first 12 hrs after the boost. Levels remained elevated until 36–48 hrs after the boost, when levels were comparable to supernatants from mock-treated explants. An asterisk indicates a significant difference from mock-transfected explants (P ≤ 0.05) at the same time point.

Since TGFβ3 is transiently expressed in the AVC endocardium [6,9], the increase in TGFβ3 levels following MBNL1 knockdown could be explained in part by a change in the timing, and not just the levels, of TGFβ3 production. For example, in the absence of MBNL1 endocardial cells may begin secreting TGFβ3 prematurely, allowing more to accumulate. Alternatively, loss of MBNL1 may allow TGFβ3 production in the endocardium to continue longer than normal. To determine when TGFβ3 levels become elevated in MBNL1-depleted explants, we performed a time course experiment (Figure 4). After a short attachment period and siRNA boost, medium was added to mock- and siRNA-transfected stage 14 AVC explants. The media were replaced with fresh medium every 12 hours until four conditioned supernatants were collected for each. Secreted TGFβ3 levels were then compared for each time point between control and MBNL1-depleted explants. TGFβ3 levels were not detectable above low baseline levels in the medium alone in control explants until the third twelve-hour window. In contrast, in MBNL1-depleted explants, TGFβ3 levels were above the baseline at all time points, and were significantly higher than those of control explants during the first 36 hours. This suggests that loss of MBNL1 not only leads to elevated levels of TGFβ3 secretion, but also precocious TGFβ3 secretion. In contrast, during the last 12 hours TGFβ3 levels went down in MBNL1-depleted supernatants and were similar to those in the control supernatants. This may suggest that MBNL1 is not required for the down-regulation of autocrine TGFβ3 production at later stages of cushion development.

**Figure 5:**
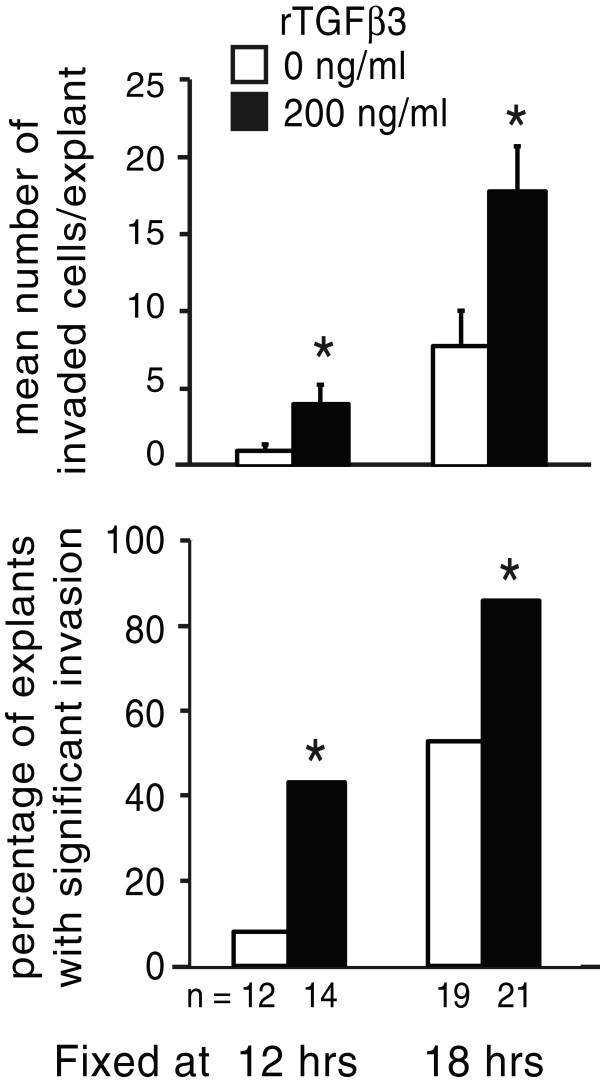
**Early exposure to elevated TGFβ3 levels induces precocious invasion.** Stage 14 AVC explants were treated with M199 supplemented with 0 or 200 ng/ml recombinant TGFβ3. The number of invaded cells was counted at 12 or 18 hours. The mean number of invaded cells per explant + the standard error of the mean is shown in the top panel; the percentage of the total number of explants that have significant invasion is shown in the bottom panel. Addition of rTGFβ3 induced an increase in the number of invaded cells per explant and the number of explants with cells undergoing invasion. An asterisk indicates a significant difference from control explants from the same stage (P ≤ 0.05).

Precocious production of TGFβ3 may in turn be predicted to promote precocious mesenchyme production. To determine whether early exposure to excess TGFβ3 will induce precocious invasion, stage 14 AVC explants were treated with recombinant TGFβ3 protein and fixed at earlier stages of culture (Figure 5). The mean number of invaded cells was significantly higher in rTGFβ3-treated explants than controls at both 12 and 18 hours. At 12 hours, only one of 12 control explants had significant levels of invasion (with four invaded cells), a fraction of the total population (8%) that does not statistically differ from an expected value of 0% by z test (P = 0.15). In contrast, over 40% of rTGFβ3-treated explants had significant numbers of invaded cells (P < 0.001 compared to a hypothetical 0% value, and P = 0.01 compared to control levels of 8%), some with ten or more invaded cells. At 18 hours, about half of control explants had significant invasion, only a few of which had more than eight invaded cells. In contrast, more than 85% of TGFβ3-treated explants had significant levels of invasion, and a third had more than 20 invaded cells. These results indicate that premature exposure to TGFβ3 does indeed induce precocious as well as an increased level of invasion.

### MBNL1 expression precedes TGFβ3 in the AVC

**Figure 6:**
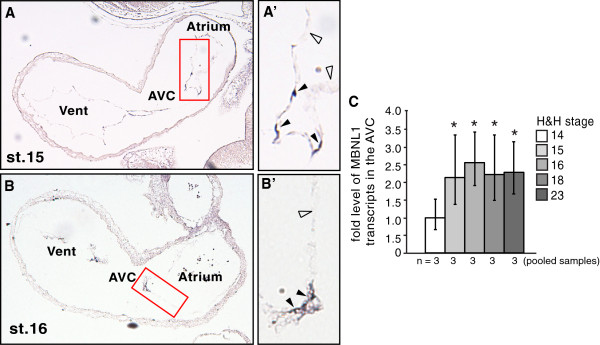
**MBNL1 is expressed in the AVC endocardium prior to EMT.** Sagittal sections of H&H stage 15 (**A**) and stage 16 (**B**) chick embryos were hybridized with *MBNL1* antisense probes. The regions indicated by red boxes are shown in higher magnification (**A’**, **B’**). Filled arrowheads indicate positively staining endocardial cells in the atrioventricular canal (AVC). Open arrowheads indicate non-expressing endocardial cells in the atrium. Positive cells in the atria and ventricles (Vent) are blood cells, which are smaller, morphologically distinct, and luminal to the endocardial lining of the chambers. (**C**) *MBNL1* transcript levels in the AVC were measured by real-time RT-PCR. Error bars denote 95% confidence intervals. An asterisk indicates a significant difference from the transcript level at stage 14 (P ≤ 0.05). Transcript levels did not differ significantly between stages 15, 16, 18, and 23 (P = 0.12 to 0.42).

If MBNL1 limits invasion by preventing precocious TGFβ3 expression in the AVC endocardium, then its expression must precede that of TGFβ3 in AVC endocardial cells. Although TGFβ3 is produced in the myocardium as early as stage 14, active TGFβ3 protein is not detectable in the AVC endocardium until stages 17–18, commensurate with EMT [6,8,9]. We previously reported that *MBNL1* transcripts are abundant in AVC endocardium at stages 18 and 23 [14], but the expression of MBNL1 prior to the onset of EMT had not been evaluated. To determine whether MBNL1 is expressed in the AVC endocardium at earlier stages, *in situ* hybridization was performed on sagittal sections from stage 15 and 16 chick embryos. Similar to what was observed at later stages [14], *MBNL1* transcripts were strongly detected in endocardial cells within the AVC at stages 15 and 16, but were low or absent in endocardial cells within the atrium and ventricle (Figure 6A, 6B). Strong *MBNL1* signal is also detected in blood cells. Adjacent sections processed in parallel and hybridized with *MBNL1* sense probes had no detectable signal (data not shown). There are far fewer AVC endocardial cells at stages 15–16 than at later stages. To determine the relative level of MBNL1 expression at different stages, the AVC region was excised from stages 14, 15, 16, 18, and 23 hearts, total RNA was harvested, and *MBNL1* levels were quantitated by real-time RT-PCR (Figure 6C). *MBNL1* transcripts were detected in the AVC at stage 14, consistent with positive staining in the heart in stage 14 embryos subjected to whole mount *in situ* hybridization [14]. The level of *MBNL1* increased at stage 15, and then remained at a steady level in the AVC through stage 23. Together, these data indicate that MBNL1 expression does indeed precede the activation of autocrine TGFβ3 expression in the AVC endocardium, consistent with our model.

### Enhancement of invasion by MBNL1 knockdown is stage-dependent in AVC explants

**Figure 7:**
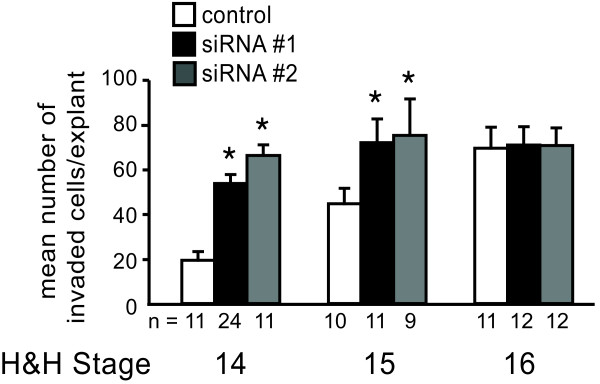
**The response to knockdown of MBNL1 is temporally regulated.** AVC explants from stage 14, 15, or 16 were transfected with or without MBNL1 siRNA and the number of invaded cells was counted at 38 hrs. The mean number of invaded cells per explant + the standard error of the mean is shown. Levels of invasion in stage 14 AVC explants treated with MBNL1 siRNA are comparable to control stage 15 explants, and levels in stage 15 AVC explants treated with MBNL1 siRNA are comparable to control stage 16 explants. Knockdown of MBNL1 did not stimulate invasion in stage 16 explants. An asterisk indicates a significant difference from control explants from the same stage (P ≤ 0.05).

To determine whether AVC endocardium from later stages would respond differently to loss of MBNL1, invasion was compared in AVC explants from stage 14, 15, and 16 transfected with control or MBNL1 siRNAs (Figure 7). As was seen previously [14], knockdown of MBNL1 with either of two independent anti-MBNL1 siRNAs in stage 14 AVC explants resulted in increased cell invasion. Knockdown in stage 15 AVC explants also enhanced invasion, whereas knockdown in stage 16 explants had no significant effect. Strikingly, the level of invasion induced by loss of MBNL1 in stage 14 explants was reminiscent of levels observed for controls at stage 15, and likewise knockdown in stage 15 explants resulted in levels similar to stage 16 controls. These results are consistent with accelerated induction of invasion. The extent of invasion in MBNL1 siRNA-treated explants from any stage did not exceed that of the normal level at stage 16. The lack of response in stage 16 explants is consistent with the normalization of TGFβ3 levels in the last window of the time course in Figure 4, and may suggest that while loss of MBNL1 stimulates precocious production of TGFβ3, once autocrine TGFβ3 is expressed MBNL1 no longer regulates its function.

### Loss of MBNL1 enhances TGFβ-dependent invasion in OFT explants

**Figure 8:**
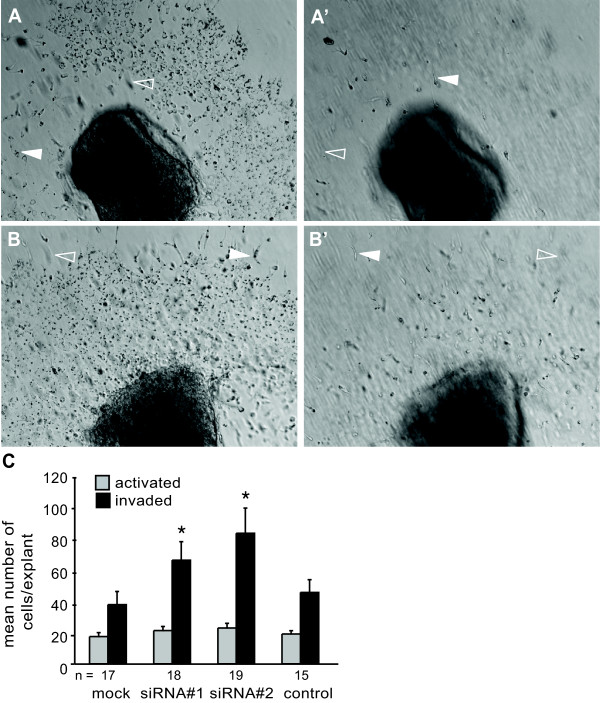
**Loss of MBNL1 stimulates endocardial cell invasion in OFT explants.** Stage 14 OFT explants were transfected with or without MBNL1 siRNA. Representative images of mock- (**A**, **A’**) or MBNL1 siRNA-treated (**B**, **B’**) OFT explants were taken in the focal plane of the surface of the gel (**A**, **B**) to show activated endocardial cells and within the gel (**A’**, **B’**) to show invaded mesenchymal cells. Filled arrowheads indicate cells in the focal plane, whereas open arrowheads indicate the same cells out of focus in a different plane. (**C**) The numbers of activated and invaded cells were counted at 38 hrs. The mean number of cells per explant + the standard error of the mean is shown. Transfection with either of two MBNL1 siRNAs, but not a control siRNA, enhanced invasion in OFT explants. Activation was not significantly affected. An asterisk indicates a significant difference from mock-transfected explants (P ≤ 0.05).

As in the AVC, endocardial cells within the OFT cushions express high levels of MBNL1 transcript [14]. To determine whether MBNL1 plays a similar role in EMT in the OFT, MBNL1 was knocked down in OFT explants from stage 14 hearts and the extent of activation and invasion were assessed (Figure 8). The results were strikingly similar to those observed in AVC explants. Either of the two independent siRNAs against MBNL1 stimulated an increase in the number of invaded cells, but had no significant effect on activation. A control siRNA had no effect on activation or invasion.

**Figure 9:**
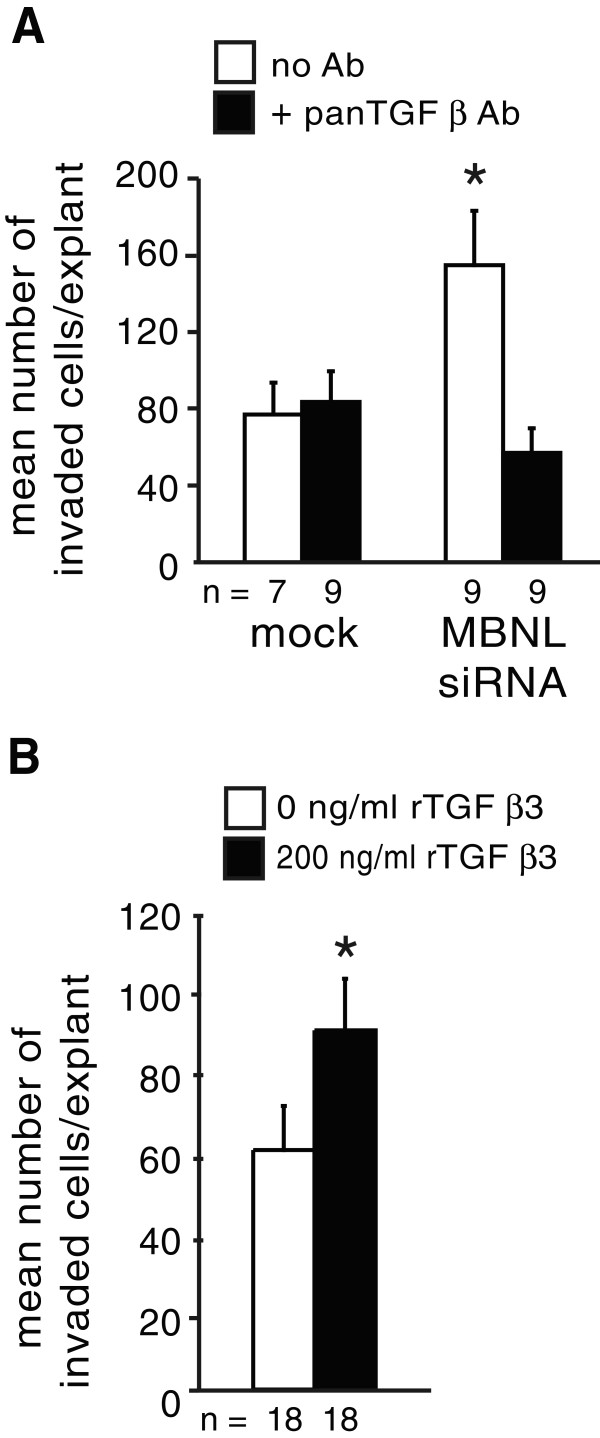
**Stimulation of cell invasion in OFT explants by knockdown of MBNL1 is TGFβ-mediated.** (**A**) Stage 14 OFT explants were transfected with or without MBNL1 siRNA. An anti-TGFβ antibody or medium alone as added at 6 hrs and again after a boost at 18 hrs. The number of invaded cells was counted at 38 hrs. The mean number of invaded cells per explant + the standard error of the mean is shown. Inhibition of TGFβ repressed the enhancement of invasion in MBNL1 siRNA-treated explants to control levels. An asterisk indicates a significant difference from mock-transfected explants without antibody treatment (P ≤ 0.05). (**B**) Stage 14 OFT explants were treated with M199 supplemented with 0 or 200 ng/ml recombinant TGFβ3. The number of invaded cells was counted at 38 hrs. The mean number of invaded cells per explant + the standard error of the mean is shown. Addition of rTGFβ3 induced an increase in cell invasion in stage 14 OFT explants. An asterisk indicates a significant difference from 0 ng/ml rTGFβ3 (P ≤ 0.05).

OFT endocardial cells and explants also express TGFβ3 coincident with active EMT [4,8,9]. To determine whether the effects of loss of MBNL1 in the OFT are also TGFβ-dependent, a pan anti-TGFβ neutralizing antibody was used to inhibit TGFβ signaling in mock- or MBNL1 siRNA-treated stage 14 OFT explants. At six hours, the anti-TGFβ antibody had no effect on the basal level of invasion in control explants, but it inhibited the enhancement of invasion following MBNL1 knockdown (Figure 9A). To determine whether supplemental TGFβ3 protein is sufficient to induce increased cell invasion in the OFT as well, stage 14 OFT explants were treated with recombinant TGFβ3 protein. As in the AVC, the addition of exogenous rTGFβ3 stimulated invasion (Figure 9B). Together, these results indicate that TGFβ is both necessary and sufficient for the enhancement of cell invasion in response to loss of MBNL1 protein in the OFT.

## Discussion

Autocrine production of TGFβ3 is required for conversion of AVC endocardial cells to invasive mesenchyme [9,10]. Furthermore, TGFβ3 is necessary and sufficient to induce expression of mesenchymal markers in chick AVC explants [6]. Here we demonstrate: (i) the ability of MBNL1 knockdown to stimulate invasion in stage 14 AVC explants is dependent on TGFβ3, (ii) endocardial-derived TGFβ3 is secreted early and at elevated levels following MBNL1 knockdown, (iii) early exposure to excess TGFβ3 induces precocious and elevated levels of mesenchymal cell invasion, and (iv) MBNL1 expression precedes that of TGFβ3 in the AVC endocardium. Together, these data support a model in which MBNL1 limits mesenchyme formation by restricting the onset and level of autocrine TGFβ3 production in the cushion endocardium. After stage 16, endocardial cells within the cushions begin to produce both active TGFβ3 protein and invasive mesenchyme [6,8,9]. Strikingly, knockdown of MBNL1 had no effect on invasion in stage 16 AVC explants, congruous with the idea that MBNL1 prevents the onset of TGFβ3 expression in the endocardium, but not its efficacy once activated.

The autocrine production of TGFβ3 in the cushion endocardium requires a signal from the myocardium [4]. Addition of exogenous TGFβ3 has been found to promote invasion in stage 14 AVC endocardial monolayers, abrogating the need for additional inductive signals from the myocardium [9,10]. We found that addition of exogenous TGFβ3 promoted the production of invasive mesenchyme in stage 14 AVC explants within 12 hours of culture. These results suggest that it is the activation of TGFβ3 expression in the endocardium, not acquisition of responsiveness to TGFβ3 signaling, that is the key event that regulates the onset of invasion. Although our model proposes that MBNL1 prevents premature activation of TGFβ3 in cushion endocardial cells, MBNL1 continues to be expressed in the AVC and OFT endocardium at stages when TGFβ3 expression and cellularization of the cushions are well underway [14]. One possibility is that a myocardial signal is required not to direct the production of TGFβ3 *per se*, but rather to alleviate its repression by MBNL1. A variety of factors secreted by the myocardium have been implicated in the induction of EMT, including TGFβs, bone morphogenetic proteins (BMPs), vascular endothelial growth factor (VEGF), hepatocyte growth factor (HGF), a 130-kDa protein (ES130) subsequently identified as a ribosome binding protein 1 homolog (RRBP1), and extracellular matrix components such as hyaluronan [5]. There is currently no known connection between any of these inductive factors and MBNL1, nor has a direct link been demonstrated between any of these and the autocrine production of TGFβ3 in the endocardium.

An important area of future investigation is to determine the mechanism by which MBNL1 prevents (and knockdown of MBNL1 thereby promotes) TGFβ3 expression in cushion endocardial cells. We found no change in the steady state levels of *TGFβ3* transcripts following MBNL1 knockdown in AVC endocardial cells. We also found no evidence of alternative splicing of *TGFβ3* transcripts (data not shown), indicating regulation of TGFβ3 occurs at the protein level. TGFβ proteins are produced and secreted in latent complexes that are activated *in vivo* by proteolytic cleavage [16]. Using antibodies that specifically recognize only active or active and latent TGFβ isoforms, one study reported that while active TGFβ3 is detectable in cushion endocardium and mesenchyme beginning at stage 17, some latent TGFβ3 is found in the AVC and OFT endocardium at stages 14–16 [8]. Latent TGFβ-binding proteins (LTBPs) are thought to be important for storage and proteolysis-mediated release of TGFβ from the latent complex, perhaps by concentrating TGFβ at the cell surface [16]. A regulatory role for LTBP-1 in endocardial cushion EMT has been identified in mice [17]. The release of active TGFβ proteins from latent complexes can be mediated by several proteases, including plasmin and thrombospondin [16]. Annexin II is expressed in stage 16 endocardial cells, and facilitates plasmin production that subsequently releases active TGFβ3 in chick AVC explants [18]. MBNL1 could act directly on the pre-mRNA alternative splicing or mRNA stability of one or more of these factors, or act indirectly on TGFβ3 activation by regulating the expression or activity of an upstream regulator of this pathway.

Although a role in TGFβ3 activation is plausible, it is also possible that loss of MBNL1 leads to an increase in protein production, secretion, or stability. In our ELISA experiments we investigated only the amount of TGFβ proteins secreted into the media, and the antibodies we used for detection in the ELISA and immunofluorescence experiments only recognize the activated TGFβ isoforms. Ongoing studies to identify the direct molecular targets of MBNL1 in the cushion endocardium will be informative in resolving the mechanism by which MBNL1 acts on TGFβ3 expression.

Although our studies have focused primarily on the role of MBNL1 in regulating endocardial cell invasion in the AVC, our data suggest MBNL1 plays a similar role in the OFT cushions. Knockdown of MBNL1 in stage 14 OFT explants enhanced invasion, and TGFβ was necessary and sufficient for this effect. Autocrine TGFβ3 is also produced by OFT endocardium, first as a latent protein and then in active form [8]. TGFβ3 has been implicated in inducing EMT during the development of other organ systems in the chick embryo as well, including fusion of the palate [19] and outgrowth of limb bud mesenchyme [20]. Strikingly, MBNL1 is expressed in the pharyngeal arches and limb buds in both mouse [21] and chicken embryos (K.R. Brimacombe and A.N. Ladd, unpublished data), raising the possibility that regulating the level and timing of TGFβ signals may be a general and conserved role of MBNL1 in the developing embryo.

## Conclusions

Autocrine TGFβ3 production is important for the formation of invasive mesenchyme from endocardial cells in the atrioventricular canal and outflow tract cushions. MBNL1 is expressed in cushion endocardial cells prior to activation of autocrine TGFβ3, and knockdown of MBNL1 leads to early and elevated levels of endocardially-derived TGFβ3 production and mesenchymal cell invasion. We therefore propose that the role of MBNL1 in the endocardial cushions is to limit the amount of invasive mesenchyme by regulating the timing and level of TGFβ3 signaling.

## Methods

### Ethics statement

Fertilized chicken eggs were purchased from Case Western Reserve University Squire Valleevue and Valley Ridge Farms and the Department of Animal Sciences at Ohio State University, so no animal husbandry was required on our part. Only early stage, pre-hatch chicken embryos were used for these studies, which are not subject to federal regulation and do not require approval from the Cleveland Clinic Institutional Animal Care and Use Committee. All embryos used in this study were taken from stages prior to the development of a differentiated nervous system, so no special measures to minimize pain and distress were needed.

### Explants, siRNA transfection, and treatments

Fertilized Babcock B-300 (Case Western Reserve University Squire Valleevue and Valley Ridge Farms) or Hy-line W-36 (Ohio State University) White Leghorn chicken eggs were incubated in 50% humidity at 100°F until desired stages were reached. Embryos were staged according to Hamburger and Hamilton [22]. Collagen gels were prepared as previously described [14].

For siRNA experiments, AVC or OFT regions were excised, transfected, and boosted with anti-MBNL1 or siGLO control siRNAs as previously described [14]. Knockdown of *MBNL1* transcripts with anti-MBNL1 siRNA is typically ~60% relative to mock-treated explants [14]. For anti-TGFβ antibody experiments, M199+ alone [1X Medium 199 (Mediatech) supplemented with 1% each ITS (Invitrogen), pen/strep antibiotic solution, and chick serum] or 10 μg/ml anti-TGFβ antibody diluted in M199+ [anti-TGF-β1, -β2, -β3 pan antibody (R&D Systems, catalog no. MAB1835), anti-TGFβ2 antibody (R&D Systems, catalog no. AB-112-NA), or anti-TGFβ3 antibody (R&D Systems, catalog no. AF-243-NA)] was added to explants. For exogenous growth factor experiments, 0, 5, 25, 50, 100, or 200 ng/ml active human recombinant TGFβ3 (R&D Systems, catalog no. 243-B3) was diluted in serum-free M199.

To measure invasion, AVC explants were fixed with 4% paraformaldehyde for 45 min at room temperature and invaded cells (i.e., cells wholly within the gel matrix) were counted using a Leica DMIRB microscope fitted with Hoffman Modulation Contrast optics. To measure EMT in OFT explants, both activated cells (i.e., isolated cells on the surface of the gel) and invaded cells were counted. When determining the percentage of explants with significant invasion, a threshold of three invaded cells was used, consistent with earlier studies [7,10,23,24]. OFT explants were imaged using QCapture Pro 6.0 software.

### Enzyme-linked immunosorbent assay (ELISA)

M199+ was added to explants and medium was collected after conditioning for 12 or 20 hours as indicated. Secreted TGFβ levels in the conditioned media were measured by sandwich ELISA. 96-well plates were coated with 2 μg/ml of a pan-TGFβ capture antibody (R&D Systems, catalog no. MAB1835) diluted in phosphate buffered saline (PBS), overnight at room temperature. Plates were washed five times with PBS + 0.05% tween-20 and blocked in PBS + 1% BSA for one hour at room temperature. After five more washes, 100 μl of sample (conditioned supernatant diluted 1:1 in PBS + 1% BSA) was added per well. A serial dilution of rTGFβ3 (R&D Systems, catalog no. 243-B3) or rTGFβ2 (R&D Systems, catalog no. 302-B2) was used to generate a standard curve. M199+ alone was diluted 1:1 in PBS + 1% BSA to establish the baseline level in unconditioned medium. Samples were incubated for two hours at room temperature. Plates were washed five times, and 0.4 μg/ml biotinylated anti-TGFβ3 (R&D Systems, catalog no. BAF243) or anti-TGFβ2 (R&D Systems, catalog no. BAF302) detection antibody diluted in PBS + 1% BSA was added. Plates were incubated two hours at room temperature, then washed five times. Streptavidin-HRP (R&D Systems, catalog no. DY-998) was diluted 1:200 in PBS + 1% BSA, added to wells, and incubated for 20 min at room temperature in the dark. Plates were washed five times, and 1-Step Slow TMB-ELISA substrate (Pierce) was added. Plates were incubated at room temperature until a strong color reaction was observed in the standards, usually about 20 min, then the reaction was stopped by addition of 1 M H_2_SO_4_. Absorbances were read at 450 nm and concentrations were calculated from the standard curve. Baseline measurements from M199+ alone were subtracted from conditioned supernatant measurements prior to comparison between experimental samples, except for values shown in Figure 4 where absolute measurements were compared directly to baseline levels in M199+ alone.

### Immunofluorescence

After 38 hrs in culture, AVC explants were fixed in 4% paraformaldehyde for 45 min at room temperature. Explants were washed twice with PBS and then incubated in permeabilization/blocking solution (3% BSA and 0.2% Triton X-100 in PBS) for 30 min at room temperature. Explants were incubated in 10 μg/mL monoclonal mouse anti-TGFβ3 (R&D Systems, catalog no. MAB463) in 3% BSA-PBS overnight at 4°C. The explants were washed once with PBS and incubated in FITC-conjugated goat anti-mouse secondary antibody (Jackson ImmunoResearch Laboratories, catalog no. 515-095-003) diluted 1:200 in 3% BSA-PBS for 2 hrs at room temperature. Explants were washed twice with PBS and counterstained (5 μg/mL Hoechst 33342 and 0.4 μg/mL DAPI in PBS) for 30 min at room temperature (Hoechst/DAPI staining not shown). Following a final wash with PBS, explants were mounted using VECTASHIELD Hard-Set Mounting Medium (Vector Laboratories) and imaged on a Leica DMIRB microscope using QCapture Pro 6.0 software.

### Real-time RT-PCR

To evaluate *TGFβ3*, *ACTA2*, and *FBN2* expression in explants, the myocardium was removed and total RNA was harvested from the remaining endocardial monolayer and endocardially-derived mesenchyme. To evaluate *MBNL1* levels during development, total RNA was harvested from AVC segments excised from stage 14, 15, 16, 18, and 23 hearts. RNA was extracted using Trizol Reagent (Invitrogen) and quantified using a NanoDrop 1000 (Thermo Scientific). At least five explants or AVC segments were pooled per sample, and each pooled sample was treated as a single biological replicate in real-time RT-PCR analyses. For each pooled sample, 1 μg RNA was converted to cDNA using the SuperScript VILO cDNA Synthesis Kit (Invitrogen) and cDNA was quantified using the Quant-iT OliGreen ssDNA Assay Kit (Invitrogen). Real-time PCR reactions were carried out using TaqMan Gene Expression Assays on the StepOnePlus platform (Life Technologies), with the following reagents: 1X TaqMan Gene Expression Master Mix, 0.25X TGFβ3 (Gg03371524_m1), 0.25X ACTA2 (Gg03352404_m1), or 0.25X FBN2 (Gg03323683_m1) FAM-labeled probe duplexed with 1X GUSB VIC-labeled probe (Gg03358465_m1; Primer Limited), and 5 ng cDNA. Each sample was run in triplicate and normalized to beta glucuronidase (*GUSB*), which did not vary in response to treatments or between developmental stages. Relative expression values for biological replicates were standardized using the method described by Willems *et al.* prior to statistical analysis [25].

### *In situ* hybridization

Embryos were fixed in 4% paraformaldehyde in PBS at 4°C overnight and dehydrated the next day by sequentially washing with 25, 50, 75, and 100% methanol in PBSw (PBS with 0.1% Tween-20). To embed, embryos were incubated in the following sequence of solutions: isopropanol at room temperature for 15 min, fresh isopropanol at 68°C for 15 min, isopropanol/paraplast (1:1) at 68°C for 30 min, liquid paraplast at 68°C twice for one hour each and once for 30 min, and solidified at room temperature. Ten micron thick sagittal sections mounted on charged slides were de-waxed and rehydrated in xylene and a 25, 50, 75, and 100% ethanol series then rinsed in 2X SSPE (300 mM NaCl, 20 mM NaH_2_PO_4_, 2 mM EDTA, pH 7.4). Slides were re-fixed in 4% paraformaldehyde in PBSw at room temperature for 15 min, rinsed in 2X SSPE, incubated in Proteinase K (3 μg/ml in PBSw) at 37°C for 30 min, and rinsed in 2X SSPE. Slides were incubated in 0.2 M HCl in PBSw at room temp for 15 min and rinsed in 2X SSPE. Slides were then incubated in a humid chamber with hybridization buffer [1% boehringer block (Roche), 50% formamide, 5X SSC (0.75 M NaCl, 0.075 M sodium citrate, pH 7.0), 12% DEPC water, 1 mg/ml Torula RNA (Roche) filtered, 0.1 mg/ml heparin in 1X SSC, 5 mM EDTA, 0.1% Tween-20, 0.1% CHAPS] at 65°C for 2 hrs after which they were incubated with RNA probe in hybridization buffer diluted 3:1000 at 65°C overnight. Sense and antisense MBNL1 probes were previously described [14]. The next day, slides were rinsed in 2X SSPE before being incubated in hybridization buffer at room temp for 5 min. Sections were then incubated in post-hybridization solution [5 ml hybridization buffer, 4.7 ml 2X SSPE, 0.3 ml 10% CHAPS] at room temp for 10 min, and then 0.3% CHAPS in 2X SSPE at room temp for 20 min. Slides were soaked in 2X SSPE at room temp for 20 min, and then rinsed with PBSw at room temp three times for 10 min each. Sections were incubated in Antibody Buffer (PBS with 10% heat inactivated goat serum, 1% boehringer block, 0.1% Tween-20) at room temp for 2 hrs. During this time, Antibody Buffer was pre-blocked with anti-dig AP fab fragments (Roche) diluted 1:1000 rocking at 4°C. Slides were then incubated in the pre-blocked Antibody Buffer at room temp for 1 hr. Sections were rinsed with 0.1% BSA in PBSw at room temp three times for 10 min each, then in AP1 Buffer (0.1 M NaCl, 0.1 M Tris pH 9.5, 50 mM MgCl_2_) at room temperature for 10 min. Slides were stained with BM Purple (Roche) at 4°C in the dark until desired strength of staining was reached. Slides were then placed in Stop Solution (100 mM Tris pH 7.4, 1 mM EDTA) at 4°C in the dark for 15 minutes before being dehydrated in a 25, 50, 75, and 100% ethanol series and xylene and finally mounted with Permount (Fisher Scientific). Sections were imaged on a Leica DM2500 microscope using QCapture Pro 6.0 software. Brightness, contrast, and color balance were adjusted using Adobe Photoshop software to match the appearance of the sections when viewed through the microscope by eye.

### Statistics

Data are reported as the mean plus the standard error of the mean unless otherwise noted. Real-time RT-PCR data are reported as the mean flanked by the upper and lower 95% confidence intervals. Comparisons between means were performed via one-tailed t-tests assuming unequal variances. Comparisons between proportions of explants with significant invasion (Figure 5) were performed via z-test. Differences were considered statistically significant when P ≤ 0.05.

## Abbreviations

MBNL1: Muscleblind-like 1; TGFβ: Transforming growth factor β; AVC: Atrioventricular canal; OFT: Outflow tract; EMT: Epithelial-mesenchymal transition; H&H: Hamburger and Hamilton.

## Competing interests

The authors declare that they have no competing interests.

## Authors’ contributions

KEL carried out the majority of explant experiments. YB-H performed immunofluorescence and real-time RT-PCR experiments, and helped with statistical analyses. SJS performed *in situ* hybridization experiments and helped with some of the explant experiments. NAV performed some of the explant experiments. ANL performed the ELISA experiments, helped with explant experiments, participated in the design and coordination of the entire study, and prepared the draft manuscript. All authors helped analyze experimental results and edit the manuscript. All authors read and approved the final manuscript.
